# Reflections on the Clinician’s Role with Individuals Who Self-identify as Transgender

**DOI:** 10.1007/s10508-021-02142-1

**Published:** 2021-09-15

**Authors:** Stephen B. Levine

**Affiliations:** Department of Psychiatry, Case Western Reserve University, 23425 Commerce Park, #104, Beachwood, OH 44122 USA

**Keywords:** Human identity, Sexual identity, Gender identity, Gender dysphoria, Ethics

## Abstract

The fact that modern patterns of the treatment of trans individuals are not based on controlled or long-term comprehensive follow-up studies has allowed many ethical tensions to persist. These have been intensifying as the numbers of adolescent girls declare themselves to be trans, have gender dysphoria, or are “boys.” This essay aims to assist clinicians in their initial approach to trans patients of any age. Gender identity is only one aspect of an individual’s multifaceted identity. The contributions to the passionate positions in the trans culture debate are discussed along with the controversy over the official, not falsifiable, position that all gender identities are inherently normal. The essay posits that it is relevant and ethical to investigate the forces that may have propelled an individual to create and announce a new identity. Some of these biological, social, and psychological forces are enumerated. Using the adolescent patient as an example, a model for a comprehensive evaluation process and its goals are provided. The essay is framed within a developmental perspective.

## Introduction

This essay derives from my clinical experiences with individuals of all ages who have sought psychiatric care for their discomfort about their gender. I have been involved with these problems since 1973. The clinical process with adults is markedly different than with younger patients. With children and adolescents, the work is fraught with greater worry about outcome, legal uncertainty concerning consent, and ethical tensions. Larger issues are in play, however, with everyone who is contemplating gender change, requesting hormones, or wanting surgery. It is increasingly recognized that the long- term mental health outcomes of these interventions are not clear. Parents, who almost always state they “want the best for their child,” look to professionals for the best management approach based on science. The trouble is that current treatment patterns are far more dependent on fashion, politically influenced beliefs, and hope. This is often referred to as best clinical practices. The media has provocatively, if not sagely, termed the controversies about gender transition as a culture war. Its debates are occurring at federal, state, and community levels and are increasingly taking place in courtrooms in the U.S. and the UK. The most common arena of intense disagreement is perhaps the least publicized one—within the family.

Many concepts about trans phenomena have evolved since the early 1970s when few mental health professionals had ever encountered a transsexual person (Pfäfflin, 2011). Psychiatric, psychological, medical, and nursing organizations continue to declare the efficacy of various forms of transition for emancipated adults. Similar pervasive endorsements did not exist for children and early adolescents where ethical concerns, policy, and science conspicuously clashed until 2018 when the American Academy of Pediatrics supported the social transition of grade school trans children and criticized watchful waiting (Rafferty, 2018): “The approach [watchful waiting] is also influenced by a group of early studies with validity concerns, methodologic flaws, and limited follow-up on children who identified as TGD [transgender and gender diverse] and, by adolescence, did not seek further treatment (“desisters”).”

Gender patients and their families often face excruciating dilemmas, some of which they feel but cannot articulately express. It is my hope that the following considerations might help clinicians to assist patients and their families with these situations.

## Gender Identity Is One Element of Individual Identity

Erikson’s (1968) body of work advanced the understanding of human development by introducing the concepts of age-expected developmental tasks, developmental lines, and virtue vs. failure labels for eight developmental phases of life. Success in the tasks of development, always individually varied because of familial and larger cultural influences, eventually created competence in the developmental lines of vocation, friendship and love, sexual behavior, stability of sexual identity, and self-management. He emphasized that how well previous tasks were accomplished determined when and how completely subsequent ones could be achieved. He proposed that psychiatric symptoms were generated by developmental failures.

Today’s concepts of identity in general, and sexual identity in particular, are far more multifaceted than Erikson’s descriptions (Stevens, 1983). Modern adult psychiatry has not emphasized adult development; it is preoccupied with symptom patterns and their treatment. Psychiatry recognizes trauma-induced dissociation, personality disorders (particularly borderline type), psychosis, and gender dysphoria as containing identity struggles. Child-oriented and psychodynamic therapists tend to be more attuned to developmental processes than those committed to treatment primarily based on DSM-5 diagnosis. The larger world understands identity in broader terms. Identity crises occur when individuals lose their jobs, retire, are widowed, get divorced, discover homosexual attraction or have their first affair, etc. There are numerous elements of human identity that can create crises. Here are some other elements: religion, ethnicity, economic status, intelligence, vocation, size (height/weight), athleticism, sense of attractiveness, sociability, political, and dietary. A consideration of an extended list of identity elements clarifies the dimensions of individuality and illuminates lives as multifaceted processes. Many of the aspects of identity are not clinically relevant at any particular time. National identity, for instance, may not be germane until one travels to another country or one is an immigrant. Racial identity heightens in the face of flagrant violations of individual rights. The number of identity elements in play at any given time is not as important a foundational concept as the idea that every person’s sense of self is multifaceted.

Most elements of identity are:

1. Associated with unique values and sensibilities
2. Passionately held
3. The source of a person’s personality development, relationships, and career
4. The explanation of individuality
5. Shapers of friendship patterns
6. Evolve from youth to old age, predictably or unpredictably, subtly or dramatically
7. Capable of enhancing, limiting, or destroying one’s life.
8. The source of emotional and physiological symptoms because of intrapsychic or interpersonal conflict
9. A cursory statement of a larger subject

### Intersectionality

One academic gender identity topic that receives considerable attention is the intersectionality of identity components (Abed et al., 2019). For example, it is well known that the physical disease and mortality risks to poor black trans females with a high school education compared to wealthy white college educated trans women are far greater in terms of HIV infection (Baral et al., 2013). But when all the elements of a person’s current identity are considered, the degree of intersectionality becomes far broader than what is usually emphasized. Intersectionality introduces the clinician to a paradox. It helps clinicians to view each trans patient as unique and to plan accordingly. When thinking about public health, political, legal, and civil rights, however, we then view trans communities as a unitary group.

## Transgender Phenomena Evoke Intense Countertransferences

Atypical gender presentations elicit strong responses in most individuals. This is perhaps less so today than decades ago, but opinions can still be intense and polarizing. Numerous factors contribute to their intensity making it difficult to discern which of these factors organize a person’s viewpoint.

1. *Fascination* with sex and gender change. The question, “Can sex be changed?” has long been explored in the arts, where men and women have for centuries been presented as the opposite sex in humor, drama, dance, opera, and popular music. Today, it is better understood that, in a basic biological sense, sex cannot be changed, but gender presentation can, with or without medical assistance.

2. *Political sensibilities*. The political Right may consider transgenderism morally wrong and dangerous to societal health. Its approach to studies and clinical services is skepticism. The political Left may consider transgenderism the courageous pursuit of self-expression, a civil right, and another praiseworthy social movement to eliminate discrimination. Its approach to studies and clinical services is positive.

3. *Religious sensibilities*. Theological assumptions may resemble either political position. In the United States, Christian religions tend to lean to the political Right.

4. *Orientation sensibilities.* Membership in the heteronormative or sexual minority communities may influence unease with, or endorsement of, transgender phenomena.

5. *Intuitive sensibilities*. When people are neither religious nor political, they may have a “gut instinct” that one should be supportive or wary of trans phenomena. Such sensibilities are best reflected through age; younger and older people have different life experiences with which to be intuitive.

Professionals overlay their attitudes from the above forces with the aspiration to be morally, politically, and religiously neutral in their clinical work. Three additional factors may also contribute to their private reactions.

1. *Personal clinical experience*. Before the evaluative gatekeeping functions of mental health professionals were downgraded in the 7th edition of the Standards of Care by WPATH (World Professional Association for Transgender Health) (Coleman et al., 2011), clinicians occasionally encountered hostile adult patients who demanded hormones or a surgery recommendation. A disrespectful hostile patient tends to create clinical wariness toward future trans individuals. With WPATH’s policy change, gender specialists became wedded to affirmation. The frequency of unpleasant clinical experiences diminished when patients were efficiently given what they desired. Nonetheless, when an affirmed patient does not do well after affirmation, a medical or surgical intervention, clinicians may hesitate more with subsequent patients,

*2. Clinical reports from innovators*. Pioneering studies of transgender treatments were compassion-based attempts to improve the lives of adults with convincing long standing painful gender/sex incongruity. The seemingly largely successful interventions were felt to not require controlled studies. Such studies might have compared predetermined comprehensively outcomes of those given hormones or a particular surgery with those who were delayed or provided psychotherapy. Conviction about the efficacy of these increasingly popular interventions made it seen to be unethical to withhold hormones and surgery to those who desired them. Individual follow-up studies that were done tended to be of short duration, with a relatively small number of patients using differing outcome measures from study to study. Many individuals in some studies were lost-to-follow data. Nonetheless, the findings encouraged gender specialists about the helpfulness of transition, hormones, and surgery. Once clinicians facilitate any form of transition, they tend to believe they are rescuing patients from endless despair and enabling happy, successful, productive lives.

3. *Knowledge of scientific studies.* Many believe that science has already firmly established the ideal way to treat gender patients. Many institutions and clinicians ignore studies that do not support their preferred concepts. For example, high desistance rates in trans children have been demonstrated in 11 of 11 studies (Cantor, 2020) but a committee of pediatricians created a policy of supporting transition of grade school children (Rafferty, 2018). One of these studies, recently published, reported follow-up of 139 Canadian boys, 63% who met criteria for gender identity disorder and 37% were subthreshold for the diagnosis. The study found that 88% had desisted at a mean age of 20.5 years (range, 13–39 years) (Singh et al., 2021). In 2020, three critiques of trans supportive interventions demonstrated that published scientific papers were flawed (Biggs, 2020; D’Angelo et al., 2021; Kalin, 2020). In 2020, a UK court put a halt to puberty blocking hormones and the use of cross-sex hormones for those under age of 16 and required 16- and 17-year-olds to individually have court approval before beginning hormones. (https://www.judiciary.uk/judgemenets/r-on-the-application-of-quincey-bell-and-a-v-tavistock-and portman-nhs-trust-and-others/). By evidence-based medicine standards (Masic et al., 2008), it has long been clear that the quality of science underlying interventions is low (Byne et al., 2012). In an objective evaluation of relevant transgender Standards of Care, WPATH’s guidelines, which clinicians have long used to justify their care, was given poor scores on relevant domains (Dahlen et al., 2021).

The interplay of the above ten factors make every clinician and family a bit uncertain what the limits of knowledge are and why this topic creates partisan passions.

## What Is Sexual Identity?

In my view, childhood and early adolescent sexual identity is incompletely formed. Later adolescent and adult sexual identity consists of gender identity, orientation, and intention components (Levine, 1989). Each of the components has internal erotic and external behavioral dimensions. Answers to three questions define an individual’s private, psychological, conscious, erotic sexual identity components: When I think of myself in terms of maleness and femaleness, who am I (gender identity)? Which gender (with biological sex assumed) attracts me romantically and sexually (orientation)? What do I want to do with a partner’s body or have done to my body during sexual activity (intention)? Erotic dimensions are not necessarily reflected in the behavioral aspects of the three components. A masculine appearing boy may privately think he is a girl. A lesbian may have heteroerotic attractions and fantasies. A sexually conventionally behaving adult may have recurrent sadomasochistic desires.

The tripartite sexual identity portion of the self is not usually appreciated in transgender evaluations, clinical care, education, or research. Sexual orientation, which WPATH asserts to be entirely distinct from gender identity (Coleman et al., 2011), has become less important in trans care because all sexual orientations are observed in trans populations and because a person’s orientation may adapt with social opportunity (Diamond et al., 2017). From its inception, most research has ignored the intention component as though it has nothing to do with the development of the sexual self and has no influence on either the origins or evolution of gender identity or orientation at any time in life (Green, 1987; Zucker et al., 2016). An unknown percentage of trans individuals have conventional intentions, but when paraphilic interests are present, the individual’s private sexual identity reflects this. A bisexual trans man who advertises himself as a submissive reflects all three dimensions of sexual identity.

## Is Transgenderism Maladaptive?

The many specific clinical, political, legal, and moral uncertainties concerning transgender phenomena ultimately stem from one underlying question: Is transgenderism an abnormality? The words “abnormality” and “psychopathology” seem to quickly move clinicians into camps and appear to some as politically incorrect (Levine & Solomon, 2009). “Is a trans identity maladaptive?” may be a somewhat less incendiary phrase. There seem to be almost three answers.

*It is not inherently maladaptive!* The official policy of the American Psychiatric Association (Byne et al., 2012) and other professional organizations is that all forms of gender identity are normal: “Being transgender or gender variant implies no impairment in judgment, stability, reliability, or general social or vocational capabilities.” Although relatively uncommon, trans binary and non-binary identities are viewed as manifestations of human developmental diversity. The argument is made that psychiatry considered gay and lesbian identities to be abnormal until 1974. Today, when this question is asked about trans people, it is skeptically viewed as the reinvention of past erroneous policies toward homosexual persons. The “No!” answer has found powerful expression in the ICD-11’s renaming the diagnosis of Gender Dysphoria as Gender Incongruence and moving it from the Mental Disorders section into Factors Affecting Sexual Health (WHO, 2018). Asymptomatic individuals who are not distressed by their unconventional gender identities bolster the argument (Askevis-Leherpeux et al., 2019). Advocates of the ICD-11 change have stated its motivation to normalize is to diminish symptoms due to self-hatred and to lessen discrimination (Reed et al., 2016). Institutional declarations of normality have not been accompanied by its evidential basis.

*It is inherently maladaptive!* For a several decades, trans phenomena were considered developmental abnormalities. The evidence was suspiciously intuitive but was also based on the high prevalence and persistence of psychiatric symptoms (Mayer & McHugh, 2016). Cross-sectional studies of these individuals, before and after social, hormonal, or surgical transitions have repeatedly affirmed this (Bränström & Pachankis, 2020a; Dhejne et al., 2016). The “Yes!” view assumes that the crystallization of trans identity is a well-intentioned, but unrealistic, solution to an unsatisfying family, interpersonal, biological, or intrapsychic environment. The new identity promises an escape—a temporary exhilarating rebirth that is fueled with intense passion. This view gains strength from health services research that describes trans populations as vulnerable groups in need of numerous protections (Liszewski et al., 2018). Data have demonstrated the need for continuing psychiatric assistance after surgical transformation (Bränström & Pachankis, 2020a, 2020b; Dhejne et al., 2011; Simonsen et al., 2016). This population’s substance abuse rates, although rarely measured in trans outcome research, is known to be significantly elevated (Compton & Jones, 2021). If persistent disabling psychiatric symptoms with frequent suicidal ideation do not qualify as maladaptive, clinicians might wonder what does? Assumptions that all trans phenomena are maladaptive solutions to prior life adverse circumstances seem too extreme when highly functional otherwise asymptomatic transgender individuals are encountered.

*It is an irrelevant question!* This view asserts that changing social roles and modifying the body of trans people is the only way to eliminate the distress of incongruence. Since these treatments cure gender dysphoria, the relevant social issues are how to increase access through insurance coverage, better education of health care professionals, and reduce discrimination (Ard & Keuroghlian, 2018). This answer seems to position the civil rights of gender diverse individuals over other considerations.

## The Natural History of Transgender Lives Is Not Known

The question for any treatment is, “How does it alter the natural course of the condition?” “Natural history” means the typical untreated course of a condition. Is the problem self-limiting, does it leave a permanent deficit, or cause early disability or death? Dramatic treatments have been offered to trans patients over a 50-year period: support through affirmation, transitioning in society, cross-sex hormones, mammoplasty or mastectomies, genital reconstruction, orchiectomy, facial feminization surgeries, and lately puberty blocking hormones. The assumption has been that these treatments create superior outcomes compared to no treatment. However, no controlled study has tested any of these treatments nor has prospectively designed follow-up studies of those undergoing treatment been accomplished (Kalin, 2020). Bränström and Pachankis’ (2020a) optimistic conclusions that claimed that transgender surgeries reduced subsequent mental health services utilization was retracted after seven critical letters to the editor and two statisticians reanalyzed their data (Bränström & Pachankis, 2020a, 2020b).

What clinicians know must be distinguished from what they believe about the natural history and the effect of treatment on the course of trans’ lives (Zucker, 2018). Psychiatric disorders are known to impair the quality of life. Clinicians should be concerned about what happens to these individuals as they move through their lives. There are only a few comprehensive national studies of this relevant question and these have worrisome results (Bränström, & Pachankis, 2020a, 2020b; Dhejne et al., 2011; Simonsen et al., 2016). In its place numerous cross-sectional studies converge on the understanding that trans communities fare less well than the general populations along many important dimensions.

Disagreements arise when these differences are explained. The minority stress hypothesis asserts that the psychiatric co-morbidities derive from social stigma that originates both in families and the culture. Young people internalize a negative attitude in the form of self-castigation, which is labeled transphobia. Transphobia is analogous to internalized homophobia within some gays and lesbians. This hypothesis is only controversial because some proponents invoke it as the only explanation of the co-morbidities. There must be other sources as well, as cis individuals also have similar but less prevalent mental problems. Incongruence itself is difficult to bear; the deeply felt aspiration to change gender expression is not fully possible, trans people have their own fears about the social, sexual functional, and relational costs of transition, and the transitions do not necessarily create lasting worry-free self-confidence in their new gender identity. When patients appear to be mentally ill or functionally impaired, clinicians confront the paradox between the policy that there is no psychopathology inherent in any trans gender form and the patient in front of them, WPATH provided a way around the paradox: “if significant physical or mental health concerns are present, they should be reasonably well controlled” (Coleman et al., 2011, p. 34). “Reasonably well controlled,” however, is a subjective appraisal by individual clinicians (Janssen et al., 2019). While professionals employ the term co-morbidity, our understanding of how they relate to one another—entirely separate or vitally interconnected—remains poor. Who can assert with clinical certainty that an individual’s symptom pattern and functional impairments have nothing to do with what the patient is trying to achieve? A survey of European endocrinologists reported ethical discomfort from not knowing the answers to fundamental questions about their adolescent patients, including “Is a trans identity a normal phenomenon?” (Vrouenraets et al., 2015). It is not unreasonable to worry about the long-term outcomes.

## Gender Dysphoria May Appear Anywhere in the Life Cycle

Some children express discomfort with their assigned gender and manifest strong cross-gender interests as early as age three. Dramatically increasing numbers of adolescents, particularly girls who never before expressed interest in or manifested cross-gender behavior are now declaring a trans identity (Nieder et al., 2011). Late adolescents and young adults who thought of themselves as gay, polyamorous, or kink may later consolidate a trans identity. Heterosexual men who long have been sexually aroused by female garments may announce that they are trans in mid-life. Inmates in male and female prisons, many of whom were bisexually behaving adolescents, are increasingly declaring themselves to be transgendered (Levine, 2016). Rarely, a person over age 60 who never recognized any homosexual or paraphilic interest or behavior changes gender. While the modal age of onset is decreasing in many countries (de Vries & Cohen-Kettenis, 2012; Eisenberg et al., 2018), gender identity, orientation, and intention can evolve throughout life (Diamond et al., 2017; Friedman & Downey, 2010; Lawrence, 2013).

## It Is Relevant to Consider “Why Now?”

Whenever trans identities emerge, many of the patients feel a drivenness to express themselves in ways that often make them and their family uneasy. Family members, clinicians, and an occasional patient ask, “Why is this occurring now?” Posing this question is a principle of good clinical management for any new symptom, for example, asking what occurred that precipitated a depressive state. Many affirmative therapists find this question to be irrelevant and unethical because of the greater need to respect patient autonomy. They may offer the idea that a trans person, at any age, knows best what is needed. It is worth recalling that today’s passion can be tomorrow’s regret. Making a diagnosis of gender dysphoria is easy. Thinking about what it is a response to is not.

## Causal Models of Gender Dysphoria (Incongruence)

Because the mechanisms of production of gender incongruence are not scientifically established, clinicians may explain the phenomena with their preferred assumptions. The ethical issue is how the clinicians’ beliefs are represented to patients, families, and colleagues.

Some assume that the cause of a trans identity is ultimately from combinations of genetics, neural development, prenatal hormones (Roselli, 2018) or post-natal environmental-induced gene expression. A more interactive nature-nurture explanation is illustrated by the elevated incidence of gender dysphoria among the autistic (Leef et al., 2019; Mahfouda et al., 2019). Autism is understood to be primarily the result of embryonic processes on brain development (Estes et al., 2019). The condition often generates social isolation, unusual interests, and rigid idiosyncratic thinking. These impairments may predispose patients to a pervasive discomfort with the self and intense degrees of loneliness, which eventually generates a hopeful search for relief through changing gender expression. A third group of explanations involve processes that may begin with poor early life bonding that leaves the youngster nervous, unconfident, or unwilling to be like a particular parent. A separation from a parent, either literally or emotionally, seems to be an inciting factor in some children and teens. At times a failure to separate from mother seems germane (Coates & Person, 1985). Trans children are over represented among runaways, those in foster care, and among adoptees (Salazar et al., 2018; Shumer et al., 2017; Zucker & Bradley, 1998). The prevalence of trans identities in prisons is much higher than in the general population (Osborne & Lawrence, 2016); inmates often have profoundly disruptive early life histories. Most child victims of egregious levels of physical, verbal, and sexual abuse and/or parental neglect do not become trans, but those who do in childhood or adulthood raise the question whether trans identity is simply an unusual outcome of common adversities.

Adolescent onset of gender dysphoria must bear some relationship to puberty when the body is changing, sexual drive appears, and awareness of social interactions and social status are heightened. One cannot discount the pervasive influence of the Internet in influencing current adolescents. Most adolescents undergo psychological strains and stresses that are only partially understandable to them. It may be possible that some adolescents who declare a trans identity are responding to ordinary developmental angst in an extraordinary manner. Some may be attracted to the cause of improving the world through expanding notions of gender. New declarations of a trans identity among early adolescents might also be understood as part of the search for how to define the self rather than the final resolution of this search (Littman, 2018). This hypothesis is in keeping with the view of adolescence that encompasses a conflictual search for a consistent sexual sense of the self (Blos, 1962; Erikson, 1968). In the light of current knowledge (Kendler, 2019), it seems unwise to assume that there is one etiology for these phenomena.

## One View of an Evaluation

While there have been reports of psychotherapeutic approaches to trans children and adolescents (Evans & Evans, 2021), the vast majority of the recent literature on management of these patients emphasizes the importance of affirmation, support for transition, diminishing internalized transphobia, and optimism about having a fulfilling life in what is labeled the authentic gender. Some advocates have declared the topic of trans care to be under researched (Winter et al., 2016). This idea relates less to the explosive number of articles being published (Sweileh, 2018), and more to the relevant unanswered questions about the efficacy of the well-known treatments and alternative approaches. The evaluation process that precedes affirmative social and medical support focuses on the criteria of the diagnosis and screening for conspicuous mental illness (Coleman et al., 2011). This represents an almost singular intense focus on gender identity as though other aspects of identity and function are irrelevant. Today, the diagnosis of gender dysphoria generates such management for older teens and adults in as little as one session, as reported to me by numerous patients and families.

Using the adolescent patient as one example, I want to discuss another approach. Any approach is modifiable based on factors such as, where the family resides, their economic resources, their structure, custody of the patient, the degree of emancipation, and previous transition. Evaluation is a bit of a misnomer as the process I am describing is also educative.

### Duration and Elements of the Process

For out of town evaluations, two days provide an ample opportunity. The evaluation begins with a joint session with patient, parents, and siblings and is followed by separate processes, including psychological testing. The first meeting with the patient generally lasts two hours, a third meeting the next day lasts an hour. Parents are seen for two hours and offered a chance to meet again the following day. Siblings are seen for an hour. We also like to have a 50-min educational session about gender dysphoria and the state of science with the family.

For in town families, the evaluation can be spread out to gain the advantage of seeing the patient and family in different mood states and after the initial anxiety somewhat dissipates. The time slots are the same, although more time can be taken with the patient and the parents if it is deemed necessary. The educational session is done prior to the final formal session for recommendations and treatment planning. The final meeting usually requires 45–60 min. The parents choose whether the siblings attend.

### Professionals Involved

One professional is the primary evaluator, with others playing defined roles. The primary evaluator and the professional who will spent the majority of time with either the patient or the parents are present at the initial and final family meetings. The initial session clarifies the family’s expectations, reviews the process, and provides an overview of sequence of events that led to the evaluation. It often reveals layers of conflict between the patient and parents and between the parents. Other therapists can meet with the siblings to ascertain their views of the family circumstances, their worries about and support of the patient. The participation of these additional professionals necessitates a professionals-only meeting to create a consensus about the relevant issues.

### Subject Matter for Inquiry

One cannot expect a teen of any age to provide a comprehensive developmental history. Parents can share the circumstances of the pregnancy, the health of the pregnancy and post partum period, and the developmental sequences of their child’s life. This includes the child’s challenges, previous physical and psychological treatments, characteristic patterns, quality of relationships with each family member, and behavioral and verbal manifestations concerning gender nonconformity. The patient can provide the vital subjective narrative about the development of gender identity and can share concepts about personal orientation and intention, sexual involvement with others, what has been learned from the Internet, fears about their trans identity, and what they desire for their future. Siblings often illuminate other relevant aspects both about parents and the patient. The goal is to reveal an accurate picture of the patient’s capacities, accomplishments, developmental challenges, symptom patterns, and motives for and degrees of gender incongruence.

### Concepts to be Communicated

Professional values are communicated to the family. Some of these are stated in the initial meeting with the family. They are repeated or introduced when it is apparent that one or more of the family members do not yet grasp them.

1. We aim to reestablish or to maintain the family’s bond to one another (in most instances). Parents and patient are not to reject one another.

2. Parents are not the trans teen’s enemy when they express concerns about their offspring’s future; they have the right and responsibility to do so. The patient may be too young, unwilling, or unable to verbalize his or her concerns about the new identity. The parents may be expressing what the patient actually thinks, but is unable to say.

3. The adolescent is in charge of determining his or her gender identity, now and in the future.

4. It is important to identify and discuss the forces that moved the teen in the direction of a new identity.

5. Humans always have ambivalence about major life changes even when they deny it.

### Types of Recommendations

We begin the final session by asking the family members how they view what has occurred. Parents are usually relieved about what has been revealed. The patients often say that the process increased their self-awareness and that they appreciate being recognized as having more dimensions than just gender identity. Most forcefully add that they are not abandoning their trans identity.

The adolescent patient and family circumstances vary considerably. The evaluation seeks to clarify many aspects of the patient’s identity as well as the patient’s past and current developmental challenges. Our recommendations begin with the identified mental health issues. These often include: separation anxiety (particularly for boys), social anxiety (most intense among those with autism), dislike of a parent or parents (particularly among divorced and chronically dysfunctional parents), depression and anxiety about peer relationships, suicidality, paraphilic excitements, substance dependence, eating disorders, other identity conflicts, and autistic traits. The idea that “I am simply trans,” is viewed as either a defense against understanding what has been going on within the person and within the family or simply a lack of understanding.

We do not recommend puberty blocking hormones and think it is prudent not to initiate cross-sex hormones prior to age 18. As many state that they desire hormones, we want to clarify what they hope the benefits will be now and in the future. We recommend an informed consent process for those seriously considering endocrine therapy. This consists of extended discussions to consider what is known about the medical, social, relational, and psychological risks of transition with or without hormones (Levine, 2019). We emphasize that this subject should not be covered quickly with a surrogate document or video. Some patients, however, come to the evaluation surreptitiously on hormones.

We recognize the vital importance of the patient’s gender identity and its impact on the future of the entire family. We want to maximize the patient’s ability to meaningfully address the identified problems and to increase the chance for success if the patient persists over time in the current gender identity. For local patients we offer our staff for psychiatric and psychotherapy services. We share our view that, despite our experience with this problem, it is the team of parents and patient, not the professional staff, who will have to discern if and when further transition is to occur. We recommend that they consider our findings at home over time. Regardless of their decisions, we ask for at least one follow-up session in three months in person, virtually, or by email.

### Final Report

If the family desires, the primary evaluator will issue a report for their use in the future. The report will have input from each of the involved professionals. It is particularly recommended for those coming from out of town.

## A Concluding Observation

The gender revolution has taught that there are more than two possible gender outcomes (Levine, 2020). The transsexuals of the 1960-1990s are increasingly the non-binary people of today (Motmans et al., 2019). Bodily discomforts and sense of not liking one’s sex have always existed in some individuals, but now there are new cultural options to deal with these discomforts. Increasing numbers of older trans adolescents and young adults are labeling themselves as genderqueer, gender fluid, pangender, third gender, hybrid gender, and more. These terms recognize gender identity as a developmental process and appreciate that some trans identified persons will not use hormones or obtain surgery (Bränström & Panchankis, 2020a; Nieder et al., 2020) and some will think of themselves as a person with a trans past (Levine, 2018; Littman, 2018). I imagine Erikson would be pleased to know that some trans people recognize he was right in emphasizing that development was a continuing process throughout life.
