# Correction to: Reflections on the Clinician’s Role with Individuals Who Self-identify as Transgender

**DOI:** 10.1007/s10508-021-02195-2

**Published:** 2021-11-01

**Authors:** Stephen B. Levine

**Affiliations:** Department of Psychiatry, Case Western Reserve University, 23425 Commerce Park, #104, Beachwood, OH 44122 USA

## Correction to: Archives of Sexual Behavior 10.1007/s10508-021-02142-1

The article “Reflections on the Clinician’s Role with Individuals Who Self-Identify as Transgender”, written by Stephen B. Levine, was originally published electronically on the publisher’s internet portal on 15 September 2021 without open access. With the author(s)’ decision to opt for Open Choice the copyright of the article changed on 6 October 2021 to © The Author(s) 2021 and the article is forthwith distributed under a Creative Commons Attribution 4.0 International License, which permits use, sharing, adaptation, distribution and reproduction in any medium or format, as long as you give appropriate credit to the original author(s) and the source, provide a link to the Creative Commons license, and indicate if changes were made. The images or other third party material in this article are included in the article's Creative Commons license, unless indicated otherwise in a credit line to the material. If material is not included in the article's Creative Commons license and your intended use is not permitted by statutory regulation or exceeds the permitted use, you will need to obtain permission directly from the copyright holder. To view a copy of this license, visit http://creativecommons.org/licenses/by/4.0/.

